# Supplementary material for: Thirteen dubious ways to detect conserved structural RNAs
Source: IUBMB Life. Author manuscript; Available in PMC 2024 Jul 10. (PMC11234323; doi:10.1002/iub.2694)
Supplement: supplementary_material [file NIHMS1907110-supplement-supplementary_material.gz › supplemental_material/rscape_v2.0.0.k.tar.gz!/rscape_v2.0.0.k/lib/R2R/R2R-current/NotByZasha/nlopt-2.6.2/src/algs/direct/userguide.pdf]

`DIRECT` Version 2.0
User Guide

J. M. Gablonsky
North Carolina State University
Department of Mathematics
Center for Research in Scientific Computation
Box 8205
Raleigh, NC 27695 - 8205

April 18, 2001

Contents

1 Introduction to DIRECT 1
1.1 Problem description 1

2 Using DIRECT 2
2.1 What is included in the package 2
2.2 Calling DIRECT 3
2.3 Sample main program 7

3 A short overview of the DIRECT algorithm and our modifications 11
3.1 Dividing the domain 12
3.1.1 Dividing of a hypercube 12
3.1.2 Dividing of a hyperrectangle 12
3.2 Potentially optimal hyperrectangles 14
3.3 The DIRECT algorithm 14
3.4 Our modification to the DIRECT algorithm 16
3.5 Extensions to the DIRECT algorithm 16

4 The Test Problems 20
4.1 Elementary functions 20
4.1.1 Constant function 20
4.1.2 Linear function 20
4.1.3 Quadratic function 20
4.2 Example for hidden constraints 20
4.2.1 Gomez #3 [11] 21
4.3 Test functions described in Jones et.al. [15] 21
4.3.1 Shekel's family (S5,S7,S10) [7] 22
4.3.2 Hartman's family (H3, H6) [7] 22
4.3.3 Branin function (BR) [7] 22
4.3.4 Goldstein and Price function (GP) [7] 24
4.3.5 Six-hump camelback function (C6) [16] 24
4.3.6 Two-dimensional Shubert function (SH) [16] 25
4.4 Main features of the Test Functions 25
4.5 Numerical results 27

Preface

This document is a revision of the 1998 guide [8] to Version 1 of our implementation of DIRECT. The major changes in the code are

- inclusion of the original DIRECT algorithm as described by Jones [15],
- extension of the algorithm to handle hidden constraints,
- and a parallel version both with PVM and MPI calls.

The code and documentation can be found at the following WWW-address :

http://www4.ncsu.edu/eos/users/c/ctkelley/www/optimization_codes.html

The primary contact for DIRECT is

J. M. Gablonsky
Department of Mathematics
Center for Research in Scientific Computations
North Carolina State University
Raleigh, NC 27695-8205
jmgablon@unity.ncsu.edu

Electronic mail is preferred.

This project was supported by National Science Foundation grants #DMS-9700569, #DMS-9714811, and #DMS-0070641, and an allocation from the North Carolina Supercomputing Center.

Owen Esslinger and Alton Patrick contributed to the parallel version of the code and the test programs.

1 Introduction to DIRECT

This user guide covers an implementation of both the original DIRECT algorithm and our modification, which we called DIRECT-l. DIRECT is a method to solve global bound constraint optimization problems and was originally developed by Jones et.al. [15]. It has been used in many industrial applications [1, 2, 4, 5, 6, 14]. We will only briefly describe our modifications to DIRECT and how we extended it to handle problems with hidden constraints and point to [9, 10] for further information.

After a short introduction in Section 1 we describe in Section 2 what is included in the package and how to use our implementation. Section 3 then gives a short explanation of the algorithm and our modifications. Finally Section 4 describes several test functions and reports some numerical results.

1.1 Problem description

DIRECT was developed to solve problems of the following form:

Problem 1 (P′) *Let* $a, b \in \mathbb{R}^N, \Omega = \{x \in \mathbb{R}^N : a_i \leq x_i \leq b_i\}$, *and* $f : \Omega \rightarrow \mathbb{R}^N$ *be Lipschitz continuous with constant* γ. *Find* $x_{opt} \in \Omega$ *such that*

$$f_{opt} = f(x_{opt}) \leq f^* + \epsilon, \tag{1}$$

where ϵ *is a given small positive constant.*

Our extension also solve more difficult problems of the following form:

Problem 2 (P″) *Let* $B \subset \Omega$ *and* $f : B \rightarrow R$ *be Lipschitz continuous with constant* γ. *Let* f^* *be*

$$f^* = \min_{x \in B} f(x).$$

Find $x_{opt} \in B$ *such that*

$$f_{opt} = f(x_{opt}) \leq f^* + \epsilon, \tag{2}$$

where ϵ *is a given small positive constant.*

If B is not given analytically, we say that the problem has hidden constraints. Problems with hidden constraints often occur in so called "black-box" optimization problems, where the objective function is given by a computer program. Note that problems of kind P″ and P′ are the same if $B = \Omega$.

2 Using DIRECT

2.1 What is included in the package

The code and documentation can be found at the following WWW-address :

http://www4.ncsu.edu/eos/users/c/ctkelley/www/optimization_codes.html

Once you have the file **DIRECTv2.0.3.tar.gz**, do the following in an UNIX environment:

unix> gunzip DIRECTv2.0.3.tar.gz

unix> tar -xf DIRECTv2.0.3.tar

If you use a computing environment other than UNIX, you may need to use other programs to uncompress the files. Once you have uncompressed the file, you will have a subdirectory called *direct* containing the following files:

DIRect.f The main routine.

DIRserial.f Special routines for serial version of DIRECT.

DIRparallel.f Special routines for parallel version of DIRECT.

DIRsubrout.f Subroutines used in DIRECT.

main.f Sample program for the serial version of DIRECT. This sample program optimizes the test functions described in Section 4.

mainparallel.f Sample program for parallel version of DIRECT.

myfunc.f Sample test functions.

DIRmpi.f Routines for parallel version of DIRECT using MPI.

DIRpvm.f Routines for parallel version of DIRECT using PVM.

makefile Makefile for sample programs (both serial and parallel).

mpi_test.cmd File to run MPI version of parallel code on the IBM SP/2 super computer.

pvm_test.cmd File to run PVM version of parallel code on the IBM SP/2 super computer.

userguide.ps This document.

To see if everything works, do the following:

unix> cd direct

unix> make

unix> TestDIRect

The sample program should solve one of the examples described below in Section 4.

Included in this package is the serial version of DIRECT as well as parallel versions both for the MPI and the PVM parallel programming standards. We used PVM 3.4 calls in the PVM version, and MPI 1.1 calls for the MPI version. We have tested both the PVM and MPI versions on the IBM SP/2 supercomputer at the North Carolina Supercomputer Center (NCSC). The files *DIRmpi.f* and *DIRpvm.f* contain interface routines to MPI and PVM, respectively, and were written by Alton Patrick.

2.2 Calling DIRECT

In this section we describe the calling sequence for DIRECT and explain the arguments following the format as in the user guide for IFFCO by Choi et.al. [3]. Finally, we provide the format for subroutines which the user must supply.

- **Calling sequence**
  Direct(fcn, x, n, eps, maxf, maxT, fmin, l, u, algmethod, Ierror, logfile, fglobal, fglper, volper, sigmaper, iidata, iisize, ddata, idsize, cdata, icsize)

- **Arguments**
  The arguments are listed in the order they appear in the calling sequence.

  - **On Entry**

fcn – is the argument containing the name of the user-supplied subroutine that returns values for the function to be minimized. *fcn* must be declared **EXTERNAL** in the calling program.

n – Integer. It is the dimension of the problem. If $n > 64$ the parameter *maxor* in the variable list at the beginning of file *DIRect.f* must be set to a larger value. *maxor* is a parameter used to dimension the work arrays used in DIRECT.

eps – Double-Precision. It ensures sufficient decrease in function value when a new potentially optimal interval is chosen. It is normally set to 10^-4, although lower values should be tried if the results of the optimization are unsatisfactory.

maxf – Integer. It is an approximate upper bound on the maximum number of function evaluations. This is only an approximate upper boundary, because the DIRECT algorithm will finish the division of all potentially optimal hyperrectangles. If it is set to a value higher than 90000, change the parameter *Maxfunc* at the beginning of file *DIRect.f*. *Maxfunc* is a parameter used to set the dimension of the work arrays used in DIRECT.

maxT – Integer. It is the maximum number of iterations. DIRECT will stop before it finishes all iterations when the maximum number of function evaluations is reached earlier. If it is set to a value higher than 600, change the parameter *Maxdeep* at the beginning of file *DIRect.f*. *Maxdeep* is used to set the dimension of the work arrays used in DIRECT.

l – Double-Precision array of length n. It defines the lower bounds for the n independent variables. The hypercube defined by the constraints on the variables is mapped to the unit hypercube in DIRECT. DIRECT performs all calculations on points within the unit cube. The final solution is mapped back to the original hypercube before being returned to the user.

u – Double-Precision array of length n. It defines the upper bounds for the n independent variables.

algmethod – Integer. It defines which method to use. The user can either use the original method as described by Jones et.al. [15] ($algmethod = 0$) or use our modification ($algmethod$

= 1). See section 3.

logfile – File-Handle for the logfile. DIRECT expects this file to be opened and closed by the user outside of DIRECT. We moved this to the outside so the user can add extra informations to this file before and after the call to DIRECT.

fglobal – Double-Precision. Function value of the global optimum. If this value is not known (that is, we solve a real problem, not a test problem) set this value to -10^{100} (or any other very large negative number) and *fglper* (see below) to 0.0.

fglper – Double-Precision. Terminate the optimization when the percent error satisfies

$$100\frac{fmin - fglobal}{\max(1, |fglobal|)} < fglper.$$

volper – Terminate the optimization once the volume of a hyperrectangle S with $f(c(S)) = f_{\min}$ is small. By small we mean that the volume of S is less than *volper* percent of the volume of the original hyperrectangle.

sigmaper – Terminate the optimization when the measure of the hyperrectangle S with $f(c(S)) = f_{\min}$ is less then sigmaper.

iidata – Integer array of length *iisize*. This array is passed to the function to be optimized and can be used to transfer data to this function. The contents are not changed by `DIRECT`.

iisize – Integer. Size of array *iidata*.

ddata – Double Precision array of length *idsize*. See *iidata*.

idsize – Integer. Size of array *ddata*.

cdata – Character array of length *icsize*. See *iidata*.

icsize – Integer. Size of array *ddata*.

– **On Return**

x – Double Precision array of length n. It is the final point obtained in the optimization process. It should be a good approximation to the global minimum for the function in the hypercube.

fmin – Double Precision. It is the value of the function at x.

Ierror – Integer. If Ierror is negative, a fatal error has occurred. The values of Ierror are as follows :

Fatal errors :

-1 *u(i)* <= *l(i)* for some i.

-2 *maxf* is too large. Increase *maxfunc*.

-3 Initialization in *DIRpreprc* failed.

-4 Error in *DIRSamplepoints*, that is there was an error in the creation of the sample points.

-5 Error in *DIRSamplef*, that is an error occurred while the function was sampled.

-6 Maximum number of levels has been reached. Increase *maxdeep*.

Successful termination :

1 Number of function evaluations done is larger then *maxf*.

2 Number of iterations is equal to *maxT*.

3 The best function value found is within *fglper* of the (known) global optimum, that is

$$100\frac{fmin - fglobal}{\max(1, |fglobal|)} < fglper.$$

Note that this termination signal only occurs when the global optimal value is known, that is, a test function is optimized.

4 The volume of the hyperrectangle with the best function value found is below *volper* percent of the volume of the original hyperrectangle.

5 The measure of the hyperrectangle with the best function value found is smaller then *sigmaper*.

- **User-Supplied Functions and Subroutines**

  – *The function evaluation subroutine*
  The name of this subroutine is supplied by the user and must be declared **EXTERNAL**. The function should have the following form (this is taken from the example in file *myfunc.f*):

```
      subroutine myfunc(x, n, flag, f, iidata, iisize,
     +                  ddata, idsize, cdata, icsize)

      implicit none
      integer n,flag,i
      double precision x(n)
      double precision f
      INTEGER iisize, idsize, icsize
      INTEGER iidata(iisize)
      Double Precision ddata(idsize)
      Character*40 cdata(icsize)

      f = 100
      do 100, i = 1,n
         f = f + (x(i)-.3)*(x(i)-.3)
100   continue
      flag = 0
      end
```

Set *flag* to 1 if the function is not defined at point *x*. The arrays *iidata, ddata* and *cdata* can be used to pass data to the function. They are not modified by DIRECT.

– *DIRInitSpecific*
This function can be found in DIRserial.f and DIRparallel.f. You can include whatever application-specific initializations you have to do in this subroutine. Most of the time you will not need it.

2.3 Sample main program

We also included a test program in the package: *main.f* for serial computers; *mainparallel.f* for parallel computers. The executables are called *TestDIRect*, *TestDIRectmpi* and *TestDIRectpvm*. This program solves 13 test problems which we describe in detail in Section 4. We also included a Matlab program that runs all these test problems with both the original DIRECT algorithm and our modification DIRECT-l. We use the following directories for this program:

direct/matlab Directory which contains the matlab program. The files contained in this directory are

- **main.m** The matlab program to run all test problems.
- **counting.m** Read in the the results from the run.
- **setdirect.m** Set the parameters for DIRECT.
- **setproblem.m** Set which problem to solve.
- **writeDIRECT.m** Write the initialization file for DIRECT.
- **writemain.m** Write the initialization file for the main program.
- **fileoutput.m** Write the results for all test problems into the file *results.txt.*

direct/ini Directory which contains the initialization files. The files contained in this directory are

- **DIRECT.ini** The file containing the parameters for DIRECT.
- **main.ini** The file containing the parameters for the main program.
- **problems.ini** The file containing the names of the initialization files for the different problems.

direct/problem Directory which contains the initialization files for the different problems.

After running *main.m*, there will be the following extra files in the main directory:

results.txt A file with a LaTeXtable listing the number of function evaluations needed and the percent error (see Section 4.5) at the end of the optimization both for DIRECT and DIRECT-l.

direct.out Log file containing information about the iterations. This file is divided into five main parts, the first and last part are generated by the user. The second part describes the parameters and some general information. The third part shows the iteration history, and the fourth part of the file gives a short summary. We now look at the structure of this file.

User data – Data written by the main program.

General information – This part first shows the version of DIRECT. In the next line we output the string stored in *cdata(1)* as the problem name. Following this we show the values of the parameters passed to DIRECT, including the bounds on the variables. We also print out if the original DIRECT algorithm or our modification is used.

Iteration history – The middle part of this file contains the iteration history. The first column contains the iteration in which DIRECT found a smaller function value than the best one known so far. The second column contains the number of function evaluations done so far, and the last column contains the best function value found. The last line of this part describes the reason why DIRECT stopped.

Summary – In the final part of this file we write out the lowest function value found, the total number of function evaluations, and how close the best function value DIRECT found is to the global minimal value, if this value is known. Furthermore we give the coordinates of the best point found and by how much these coordinates differ from the upper and lower bounds.

User data – Additional data written by the main program.

Below we show an example for this file created by the sample program included in the package:

User data

```
+-----------------------------------------+
|       Example Program for DIRECT        |
|  This program uses DIRECT to optimize   |
|  testfunctions. Which testfunction is   |
| optimized and what parameters are used  |
| is controlled by the files in ini/.     |
|                                         |
|     Owen Esslinger, Joerg Gablonsky,    |
|             Alton Patrick               |
|              04/15/2001                 |
+-----------------------------------------+
Name of ini-directory    : ini/
Name of DIRect.ini file  : DIRECT.ini
```

```
Name of problemdata file : shekel5.ini
Testproblem used         :    5
```

General information

```
-------------------------------- Log file --------------------------------
DIRECT Version 2.0.3
 Shekel-5 function
 Problem Dimension n                          :      4
 Eps value                                    :   0.1000E-03
 Maximum number of f-evaluations (maxf)       :  20000
 Maximum number of iterations (MaxT)          :   6000
 Value of f_global                            :  -0.1015E+02
 Global percentage wanted                     :   0.1000E-01
 Volume percentage wanted                     :  -0.1000E+01
 Measure percentage wanted                    :  -0.1000E+01
 Epsilon is constant.
 Jones original DIRECT algorithm is used.
Bounds on variable x 1    :      0.00000 <= xi <=     10.00000
Bounds on variable x 2    :      0.00000 <= xi <=     10.00000
Bounds on variable x 3    :      0.00000 <= xi <=     10.00000
Bounds on variable x 4    :      0.00000 <= xi <=     10.00000
```

Iteration history

```
---------------------------------------------------------------------------
 Iteration   # of f-eval.   fmin
    1           9          -0.5753514094
    3          43          -0.6989272350
    4          51          -1.0519854213
    5          57          -6.8404676192
    7          81          -7.4383120011
    8          91          -8.1524902009
    9          99          -9.0180871080
   10         103         -10.0934485966
   12         129         -10.1082368755
   13         143         -10.1230718067
```

```
   14         151         -10.1376865940
   15         155         -10.1523498373
DIRECT stopped: fmin within fglper of global minimum.
```

Summary

```
-------------------------------- Summary --------------------------------
Final function value           :  -10.1523498
Number of function evaluations :         155
Final function value is within    0.00837 percent of global optimum.
Index  Final solution   x(i) - l(i)   u(i) - x(i)
    1    3.9986283       3.9986283      6.0013717
    2    3.9986283       3.9986283      6.0013717
    3    3.9986283       3.9986283      6.0013717
    4    3.9986283       3.9986283      6.0013717
-------------------------------------------------------------------------------
```

User data

```
-------------- Final result ------------------
DIRECT termination flag :   3
DIRECT minimal point    :    3.9986283   3.9986283   3.9986283   3.9986283
DIRECT minimal value    :  -10.1523498
DIRECT number of f-eval :   155
Time needed             : 0.3000E-01 seconds.
```

3 A short overview of the DIRECT algorithm and our modifications

In this section we give a mathematical description of the original DIRECT algorithm, which was developed by D. R. Jones, C. D. Perttunen and B. E. Stuckman [15] in 1993, and our modifications to it. The name DIRECT is derived from one of its main features, *di*viding *rect*angles. There are two main ingredients to this algorithm. The first is how to divide the domain (Section 3.1), and the second ingredient is how to decide which hyperrectangles we divide in the next iteration (Section 3.2).

3.1 Dividing the domain

Division is based on N-dimensional trisection. Sections 3.1.1 and 3.1.2 describe how this division is done for a hypercube and a hyperrectangle , respectively.

3.1.1 Dividing of a hypercube

Let c be the center point of a hypercube. The algorithm evaluates the function at the points $c \pm \delta e_i$, where δ equals 1/3 of the side length of the cube and e_i is the i-th Euclidean base vector. DIRECT defines w_i by

$$w_i = \min\{f(c + \delta e_i), f(c - \delta e_i)\}.$$

The algorithm then divides the hypercube in the order given by the w_i, starting with the lowest w_i. DIRECT divides the hypercube first perpendicular to the the direction with the lowest w_i. Then it divides the remaining volume perpendicular to the direction of the second lowest w_i and so on until the hypercube is divided in all directions. This strategy puts c in the center of a hypercube with side length δ. Let $b = \arg\min_{i=1,\ldots,N}\{f(c+\delta e_i), f(c-\delta e_i)\}$. b will be the center of a hyperrectangle with one side with a length of δ, the other $N-1$ sides will have a length of 3δ.

Figure 1a shows an example of the division of a hypercube. Here

$$\begin{aligned} w_1 &= \min\{5, 8\} = 5 \\ w_2 &= \min\{6, 2\} = 2. \end{aligned}$$

Therefore we divide first perpendicular to the x_2-axis, and then in the second step the remaining rectangle is divided perpendicular to the x_1-axis.

3.1.2 Dividing of a hyperrectangle

In DIRECT a hyperrectangle is only divided along its longest sides, which assures us that we get a decrease in the maximal side length of the hyperrectangle.

Figure 1b represents the next step in the algorithm. DIRECT will divide the shaded area (We explain in Section 3.2 how we choose which hyperrectangles to divide). The second box in Figure 1b shows where DIRECT samples the function, and the third box shows how the rectangle is only divided once.

a
b
c
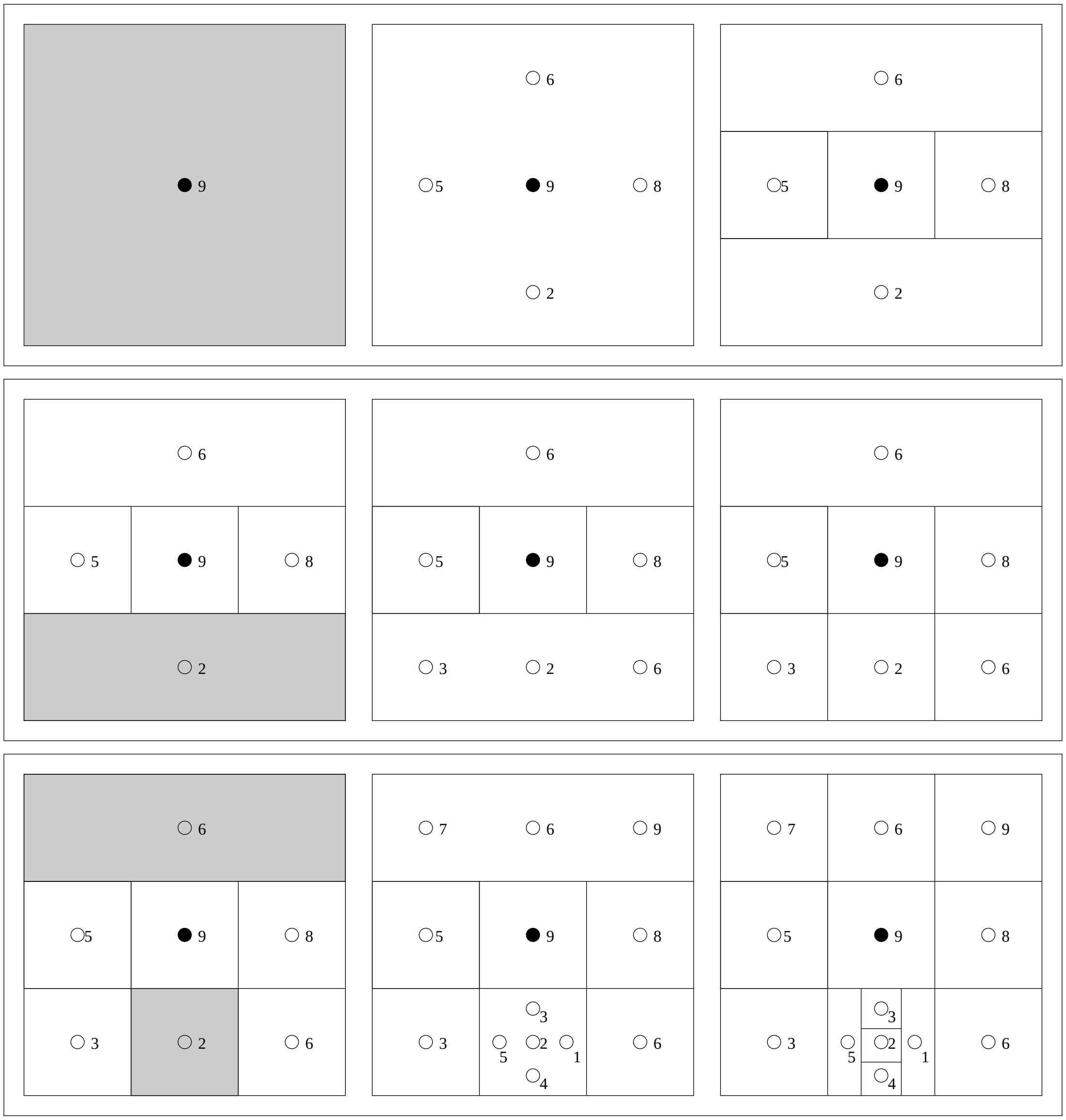

Figure 1: Dividing of a hypercube.

Figure 1c shows the third step in the algorithm for this example. In this step DIRECT will divide two rectangles, which are shaded. One of them is a square, therefore it is divided twice as described before. The larger area is again a rectangle and gets divided once.

3.2 Potentially optimal hyperrectangles

This section describes the second main ingredient for the DIRECT algorithm, how to decide which hyperrectangles to divide in the next iteration. DIRECT divides all potentially optimal hyperrectangles as defined in definition 1.

Definition 1 *Let $\epsilon > 0$ be a positive constant and let f_{min} be the current best function value. A hyperrectangle j is said to be potentially optimal if there exists some $\tilde{K} > 0$ such that*

$$\begin{aligned} f(c_j) - \tilde{K} d_j &\leq f(c_i) - \tilde{K} d_i, \ \forall i, \ \textit{and} \\ f(c_j) - \tilde{K} d_j &\leq f_{min} - \epsilon |f_{min}|. \end{aligned}$$

In this definition c_j is the center of the hyperrectangle j, and d_j is a measure for this hyperrectangle. Jones et.al. [15] chose d_i to be the distance from the center of hyperrectangle i to its vertices. They divide all potentially optimal hyperrectangles in every iteration, even if two of them have the same measure and the same function value at the center.

3.3 The DIRECT algorithm

We give a formal description of the DIRECT algorithm in algorithm 1.

The first two steps in the algorithm are the initialization steps. The variable m is a counter for the number of function evaluations done while t is a counter for the number of iterations. Unlike more traditional optimization methods, there is no termination criteria based on the function for DIRECT. Instead DIRECT stops after $numit$ iterations or after $numfunc$ function evaluations. Note that the limit on the number of function evaluations is not strictly enforced. We only check for this condition after we have divided all potentially optimal hyperrectangles in an iteration. This means we normally do a few more function evaluations than $numfunc$.

Note that in algorithm 1 there are two possibilities of parallelism here. These are the inner loop (steps 5 to 10) and the function evaluations inside

Algorithm 1 DIRECT$(a, b, f, \epsilon, numit, numfunc)$

1: Normalize the search space to be the unit hypercube with center point c_1
2: Evaluate $f(c_1), f_{min} = f(c_1), t = 0, m = 1$
3: **while** $t < numit$ and $m < numfunc$ **do**
4: Identify the set S of potentially optimal hyperrectangles
5: **while** $S \neq \emptyset$ **do**
6: Take $j \in S$
7: Sample new points, evaluate f at the new points and divide the hyperrectangle with **Divide**
8: Update $f_{min}, m = m + \Delta m$
9: Set $S = S \setminus \{j\}$
10: **end while**
11: $t = t + 1$
12: **end while**

the inner loop (step 8). In our parallel implementation only the inner loop is parallelized.

Potentially optimal intervals are identified by DIRECT using the following Lemma to reformulate Definition 1.

Lemma 1 *Let $\epsilon > 0$ be a positive constant and let f_{min} be the current best function value. Let I be the set of all indices of all intervals existing. Let $I_1 = \{i \in I : d_i < d_j\}$, $I_2 = \{i \in I : d_i > d_j\}$ and $I_3 = \{i \in I : d_i = d_j\}$. Interval $j \in I$ is potentially optimal if*

$$f(c_j) \leq f(c_i), \forall i \in I_3, \tag{3}$$

there exists $\tilde{K} > 0$ such that

$$\max_{i \in I_1} \frac{f(c_j) - f(c_i)}{d_j - d_i} \leq \tilde{K} \leq \min_{i \in I_2} \frac{f(c_i) - f(c_j)}{d_i - d_j}, \tag{4}$$

and

$$\epsilon \leq \frac{f_{\min} - f(c_j)}{|f_{\min}|} + \frac{d_j}{|f_{\min}|} \min_{i \in I_2} \frac{f(c_i) - f(c_j)}{d_i - d_j}, \ f_{\min} \neq 0, \tag{5}$$

or

$$f(c_j) \leq d_j \min_{i \in I_2} \frac{f(c_i) - f(c_j)}{d_i - d_j}, \ f_{\min} = 0. \tag{6}$$

The proof of this lemma can be found in [9].

3.4 Our modification to the DIRECT algorithm

In our modification we use the length of the longest side of a hyperrectangle as the measure d_j. This reduces the number of different groups of hyperrectangles compared to using the distance from the center to a corner, and makes the algorithm more biased towards local search.

The second modification we did was to divide at most one hyperrectangle per group. That is, if there is more than one hyperrectangle with the same measure, we divide only one of them, instead of all. This again can lead to an improvement of the performance of the algorithm. Both these modifications together result in DIRECT-l.

3.5 Extensions to the DIRECT algorithm

We also extended the algorithm to handle problems with hidden constraints. That means we look at Problems P$''$ where the subset $B \subset \Omega$ is not given analytically. If no feasible point is found within the budget given to DIRECT we allow it to continue until a feasible point is found and then reassign the original budget. Through this strategy we assure that DIRECT does not terminate without finding a feasible point.

The strategy we use was suggested by R. Carter [1]. We describe the general idea before going into details.

For any infeasible midpoint, we expand its hyperrectangle by a factor of two. If this larger hyperrectangle contains one or more feasible midpoints of other hyperrectangles, find the smallest function value of these, f_{min}. Then use $f_{min} + \epsilon|f_{min}|$ as a surrogate value. If no feasible midpoint is contained in the larger hyperrectangle, mark the current point as really infeasible.

We will now describe this strategy in more details.

We extend DIRECT as shown in Algorithm 2 by adding a call to **ReplaceInf** in line 3.5. In this method the actual replacement takes place.

In method **ReplaceInf**, shown in Algorithm 3, we iterate over all hyperrectangles with infeasible midpoints. For each of these midpoints, we create a new surrounding box by doubling the length of each side while keeping the same center. Then we find f_{minloc}, which is the minimum value of all feasible points calculated by DIRECT inside this expanded hyperrectangle . If this minimum exists (that is, there is at least one feasible point in the larger hyperrectangle) we assign $f_{minloc} + \epsilon|f_{minloc}|$ to the current infeasible point. We used a value of $\epsilon = 10^{-6}$ in our computations. Otherwise the infeasible

Algorithm 2 DIRECT$(a, b, f, \epsilon, numit, numfunc)$

Normalize the search space to be the unit hypercube with center point c_1
Evaluate $f(c_1), f_{min} = f(c_1), t = 0, m = 1$
while $t < numit$ and $m < numfunc$ **do**
  Identify the set S of potentially optimal hyperrectangles
  while $S \neq \emptyset$ **do**
    Take $j \in S$
    Sample new points, evaluate f at the new points and divide the hyper-rectangle with **Divide**
    Update $f_{min}, m = m + \Delta m$
    Set $S = S \setminus \{j\}$
  end while
  Use **ReplaceInf** to check for infeasible points which are near feasible points and to replace the value at these by the value of a nearby point.
  $t = t + 1$
end while

point is marked really infeasible.

We assign the maximum value found so far, increased by 1, to really infeasible points. Since we have to check each infeasible point in **ReplaceInf** (see below) there is no extra cost if the maximum increases.

We increase the replacement value to make sure that if there is another hyperrectangle with the same measure, feasible midpoint, and the same function value (this could be one we used to calculate the minimum), the one with feasible midpoint is divided first.

Figure 2 shows an example of this strategy. We show a close-up of the area around an infeasible point P and its corresponding hyperrectangle. The dotted rectangle is the enlarged rectangle around P. There are nine midpoints of rectangles created by DIRECT contained in this enlarged rectangle; three of them are infeasible. Note that we look at the closed rectangle, therefore the points on the boundary are also considered nearby. Therefore, we only need to take the minimum over the other six feasible midpoints. This value is given by 10; therefore we take $10 + \epsilon|10|$ as the new value at P. If we would not amplify the surrogate value, the rectangle with center P would look (to DIRECT) the same as the rectangle with a function value of 10 at

Algorithm 3 ReplaceInf($\{c_i\}, \{l_i\}, \{f_i\}$)

Input :

- $\{c_i\}$ - centers of hyperrectangles created by DIRECT, $c_i \in \mathbb{R}^N$.
- $\{l_i\}$ - side lengths of the hyperrectangles created by DIRECT, $l_i \in \mathbb{R}^N$.
- $\{f_i\}$ - function values at centers of hyperrectangles created by DIRECT, $f_i \in \mathbb{R}$.

Output :

- $\{f_i\}$ - updated function values.

for all c_i infeasible **do**
    Create larger hyperrectangle D around c_i.
    $F = \min\{\min_{c_j \in D} f_j, \infty\}$
    if $F < \infty$ **then**
        $f_i = F + 10^{-6}|F|$
    else
        mark f_i really infeasible.
    end if
end for

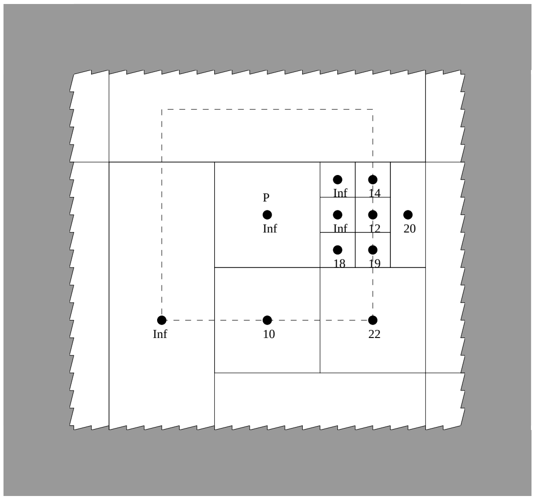

Figure 2: Example of an infeasible point and how its surrogate value is calculated.

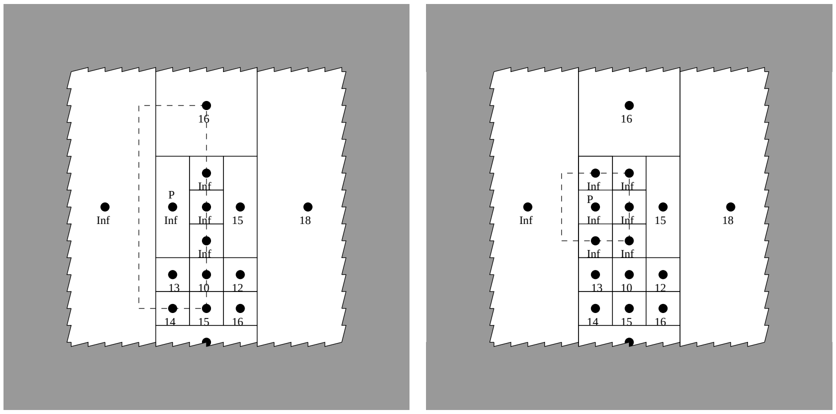

Figure 3: Example of an infeasible point, whose value was replaced by the value of a feasible point nearby, becoming completely infeasible.

the midpoint. Therefore, we would have to ensure that DIRECT chooses the rectangle with feasible midpoint and not the one with midpoint P. We avoid this problem by using the amplified value.

Note that this surrogate value at the midpoint can change in each outer iteration. There are two reasons why this could happen.

- A new point inside the box with a lower function value than the one assigned so far has been found.
- The hyperrectangle corresponding to the infeasible point was divided by DIRECT. Through this division DIRECT has made the hyperrectangle smaller and no more feasible point is nearby (that is in the area looked at).

Figure 3 shows an example of this. On the left, we show a close-up of the hyperrectangles and midpoints created by DIRECT before the call to **ReplaceInf**. We show the enlarged rectangle around P. There are eight points inside this enlarged rectangle (including the boundary). Five of these are feasible points; therefore, we assign the surrogate value of $10 + \epsilon|10|$ to the point P.

On the right of Figure 3 we show the same area after DIRECT has divided the rectangle corresponding to P. The two newly created rectangles have infeasible midpoints. This time the enlarged rectangle around P does not contain any feasible points (at least DIRECT has not found any). Therefore P is now marked as really infeasible.

4 The Test Problems

We first give short descriptions of all the test functions we looked at. Following the descriptions we summarize the important features of the functions and describe (shortly) our numerical results. These can be redone easily with the package provided.

4.1 Elementary functions

The first three functions we look at are examples of constant, linear and quadratic functions. Looking at the behavior of DIRECT for these functions allows us to get a better understanding of DIRECT and shows the differences between the original algorithm and our modification. The functions are

4.1.1 Constant function

$$f(x) = 100, \quad \Omega = [0,1]^N.$$

In the example program we set $N = 2$.

4.1.2 Linear function

$$f(x) = 2x_1 + \sum_{i=2}^{N} x_i, \quad \Omega = [0,1]^N.$$

In the example program we set $N = 2$. The optimal point $x^* = (0, \ldots, 0)^T$ has an optimal function value of $f^* = 0$.

4.1.3 Quadratic function

$$f(x) = 10 + \sum_{i=1}^{N} (x_i - 5.3)^2, \quad \Omega = [0,10]^N.$$

In the example program we set $N = 2$. The optimal point $x^* = (5.3, \ldots, 5.3)^T$ has an optimal function value of $f^* = 10$.

4.2 Example for hidden constraints

The following test function comes from Jones [14]. It was originally given in Gomez et.al. [11].

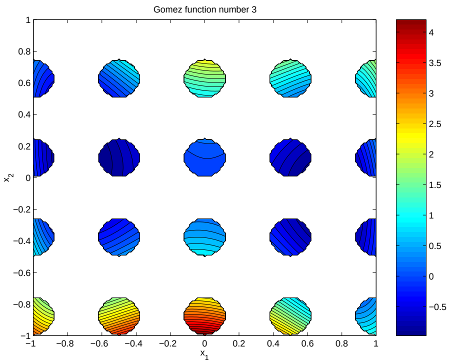

Figure 4: Contour plot of the Gomez # 3 function.

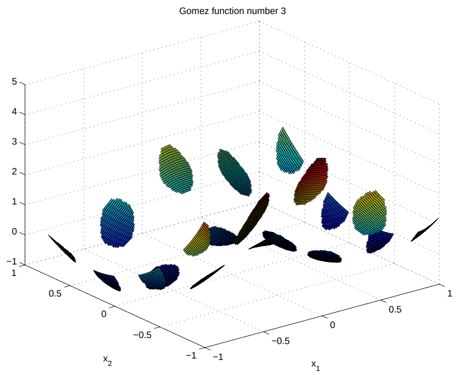

Figure 5: Plot of the Gomez # 3 function.

4.2.1 Gomez #3 [11]

$$\begin{aligned} f(x) &= \left(4 - 2.1x_1^2 + \frac{x_1^4}{3}\right) x_1^2 + x_1 x_2 + 4(x_2^2 - 1)x_2^2, \\ \Omega &= [-1, 1]^2, \\ B &= \Omega \cap \{x \in \mathbb{R}^2 | -\sin(4\pi x_1) + 2\sin(2\pi x_2) \leq 0\} \end{aligned}$$

We used this problem as if we did not know the nonlinear constraints. Whenever the nonlinear constraint was not satisfied, we returned $flag = 1$. The function has a minimum at $x^* = (0.109, -0.623)^T$ with a function value of $f^* = -0.9711$. In figure 4 we show a contour plot of this function. It is clear that the domain of this function consists of several disconnected regions. We show a plot of the function in figure 5.

4.3 Test functions described in Jones et.al. [15]

Jones et al. [15] describe results for their original implementation of DIRECT on nine different test problems. The first seven problems were originally given by Dixon and Szegö [7]. These problems have been widely used to compare global optimization algorithms [7, 12, 13]. Problems eight and nine come from Yao [16]. We now describe these test problems in more detail.

Table 1: Parameters for the Shekel's family of functions

i	a_i^T				c_i
1	4.0	4.0	4.0	4.0	.1
2	1.0	1.0	1.0	1.0	.2
3	8.0	8.0	8.0	8.0	.2
4	6.0	6.0	6.0	6.0	.4
5	3.0	7.0	3.0	7.0	.4
6	2.0	9.0	2.0	9.0	.6
7	5.0	5.0	3.0	3.0	.3
8	8.0	1.0	8.0	1.0	.7
9	6.0	2.0	6.0	2.0	.5
10	7.0	3.6	7.0	3.6	.5

4.3.1 Shekel's family (S5,S7,S10) [7]

$$f(x) = -\sum_{i=1}^{m} \frac{1}{(x-a_i)^T(x-a_i)+c_i}, x, a_i \in \mathbb{R}^N, c_i > 0, \forall i = 1,\ldots,m,$$

$$\Omega = [0,10]^N.$$

Three instances of the Shekel function are used in the comparisons. Here $N = 4, m = 5, 7$ and 10. The values of a_i and c_i are given in Table 1.

4.3.2 Hartman's family (H3, H6) [7]

$$f(x) = -\sum_{i=1}^{m} c_i \exp\left(-\sum_{j=1}^{N} a_{ij}(x_j - p_{ij})^2\right), x, a_i, p_i \in \mathbb{R}^N, c_i > 0, \forall i = 1,\ldots,m,$$

$$\Omega = [0,1]^N.$$

We will look at two instances of the Hartman function. The values of the parameters and the dimensions of the problems are given in Table 2.

4.3.3 Branin function (BR) [7]

$$f(x_1,x_2) = (x - 2 - \frac{5.1}{4\pi^2}x_1^2 + \frac{5}{\pi}x_1 - 6)^2 + 10(1-\frac{1}{8\pi})\cos x_1 + 10,$$
$$\Omega = [-5,10]\times[0,15].$$

Table 2: Parameters for the Hartman's family of functions

First case : $N = 3, m = 4$.

i	a_i			c_i	p_i		
1	3.	10.	30.	1.	0.3689	0.1170	0.2673
2	.1	10.	35.	1.2	0.4699	0.4387	0.7470
3	3.	10.	30.	1.	0.1091	0.8732	0.5547
4	.1	10.	35.	3.2	0.03815	0.5743	0.8828

Second case : $N = 6, m = 4$.

i	a_i						c_i
1	10.	3.	17.	3.5	1.7	8.	1.
2	.05	10.	17.	.1	8.	14	1.2
3	3.	3.5	1.7	10.	17.	8.	3.
4	17.	8.	.05	10.	.1	14.	3.2

i	p_i					
1	0.1312	0.1696	0.5569	0.0124	0.8283	0.5886
2	0.2329	0.4135	0.8307	0.3736	0.1004	0.9991
3	0.2348	0.1451	0.3522	0.2883	0.3047	0.6650
4	0.4047	0.8828	0.8732	0.5743	0.1091	0.0381

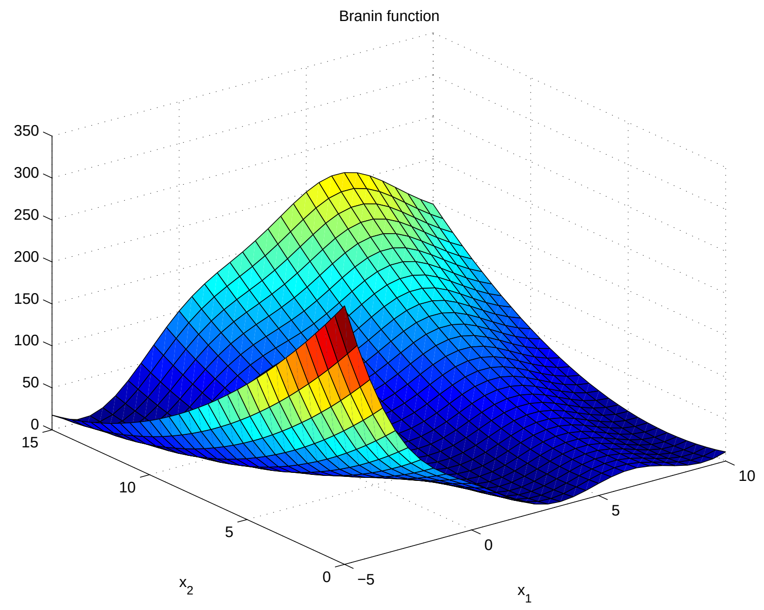

Figure 6: Plot of the Branin function.

This function has three global minima. Figure 6 shows a plot of this function.

4.3.4 Goldstein and Price function (GP) [7]

$$
\begin{aligned}
f(x_1, x_2) &= \left[1 + (x_1 + x_2 + 1)^2(19 - 14x_1 + 3x_1^2 - 14x_2 + 6x_1x_2 + 3x_2^2)\right] \\
&\quad \left[30 + (2x_1 - 3x_2)^2(18 - 32x_1 + 12x_1^2 + 48x_2 - 36x_1x_2 + 27x_2^2)\right], \\
\Omega &= [-2, 2]^2.
\end{aligned}
$$

The function has four local minima and one global minimum at $x^* = (0, -1)^T$ with $f(x^*) = 3$. In figures 7 and 8 we show plots of this function. The first figure shows the whole domain, while the second figure shows only the area around the global minimum.

4.3.5 Six-hump camelback function (C6) [16]

$$
\begin{aligned}
f(x_1, x_2) &= (4 - 2.1x_1^2 + x_1^4/3)x_1^2 + x_1x_2 + (-4 + 4x_2^2)x_2^2, \\
\Omega &= [-3, 3] \times [-2, 2].
\end{aligned}
$$

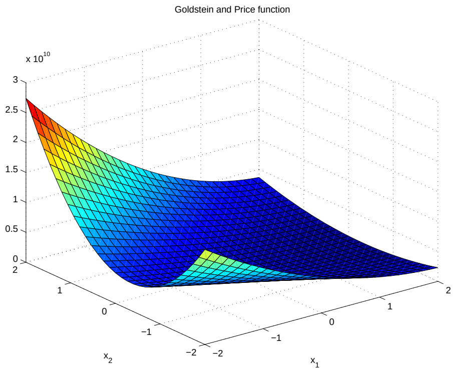

Figure 7: Plot of the Goldstein and Price function.

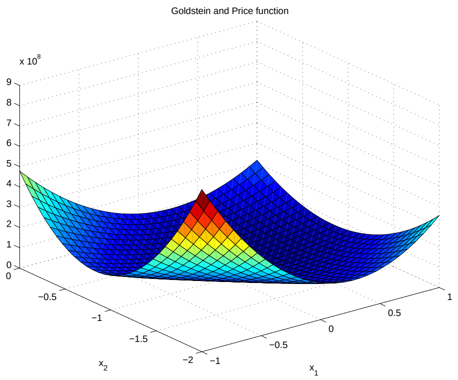

Figure 8: Plot of the Goldstein and Price function around the global minimum.

The function has six minima, two of which are global. The global minima are located at $x^* = (\pm 0.0898, \mp 0.7126)^T$ and $f(x^*) = -1.0316$. Figures 9 and 10 show plots of this function. Again, the first figure shows the whole domain, while the second shows an enlargement around the global minima.

4.3.6 Two-dimensional Shubert function (SH) [16]

$$f(x_1, x_2) = \left(\sum_{j=1}^{5} j \cos[(j+1)x_1 + j] \right) \left(\sum_{j=1}^{5} j \cos[(j+1)x_2 + j] \right),$$
$$\Omega = [-10, 10]^2.$$

The function has 760 local minima, of which 18 are global. Figures 11 and 12 show two plots of this function. Again, the first figure shows the whole domain, while the second shows an enlargement around one of the global minima.

Table 3 summarizes the important features of the test problems used in our numerical experiments.

4.4 Main features of the Test Functions

In table 3 we give the box constraints, the number of global minima and the function values at the global minima for the test functions described above.

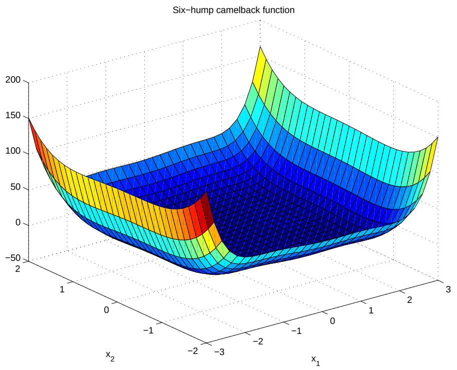

Figure 9: Plot of the Six-hump camelback function.

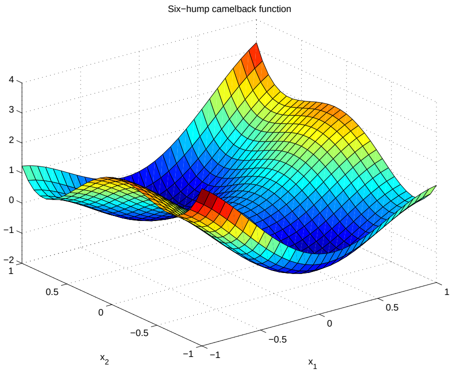

Figure 10: Plot of the Six-hump camelback function around the global minima.

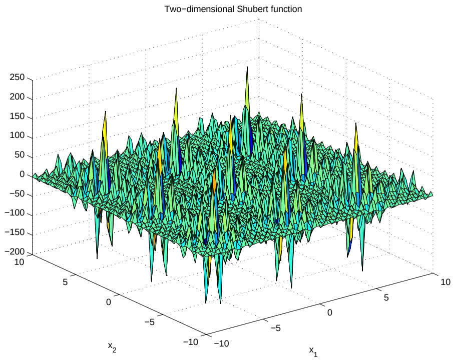

Figure 11: Plot of the two-dimensional Shubert function.

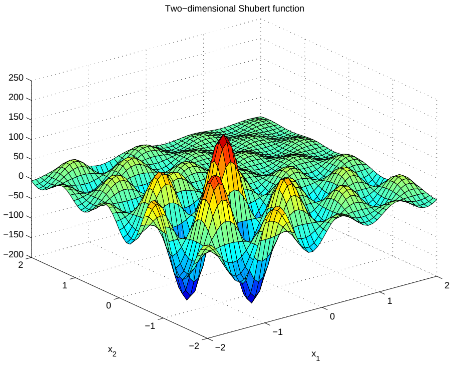

Figure 12: Plot of the two-dimensional Shubert function around a global minimum.

Table 3: Summary of the important features of the test functions.

#	Name	N	Ω	Global minima number	Global minima function value
1	Constant	2	$[0,1]^2$	∞	100
2	Linear	2	$[0,1]^2$	1	0
3	Quadratic	2	$[0,10]^2$	1	10
4	Gomez 3	2	$[-1,1]^2$	1	-0.981243
5	Branin (BR)	2	$[-5,10] \times [0,15]$	3	0.398
6	Shekel-5 (S5)	4	$[0,10]^4$	1	-10.153
7	Shekel-7 (S7)	4	$[0,10]^4$	1	-10.403
8	Shekel-10 (S10)	4	$[0,10]^4$	1	-10.536
9	Hartman-3 (H3)	3	$[0,1]^3$	1	-3.863
10	Hartman-6 (H6)	6	$[0,1]^6$	1	-3.322
11	Goldprice (GP)	2	$[-2,2]^2$	1	3.000
12	Sixhump (C6)	2	$[-3,3] \times [-2,2]$	2	-1.032
13	Shubert (SH)	2	$[-10,10]^2$	18	-186.831

4.5 Numerical results

Table 4 reports the results for `DIRECT` and `DIRECT-l` using the termination criteria from Jones et al. [15]. This termination criteria uses knowledge of the global minimum value to terminate once the percentage error is small. For this we first define the percentage error.

Let f_{global} be the known global minimal function value and denote by f_{min} the best function value found by `DIRECT`. We define the *percentage error* p as

$$p = \begin{cases} 100\frac{f_{min} - f_{global}}{|f_{global}|}, & f_{global} \neq 0, \\ 100 f_{min}, & f_{global} = 0. \end{cases}$$

Following Jones, we terminate the iteration once p is lower than 0.01. In all runs we set $\epsilon = 0.0001$ for both the original implementation and our implementation.

Although both methods can solve this problem, our modification consistently requires fewer function evaluations, significantly fewer for problems 2 through 3 and 9 through 13. This means the algorithm terminated with $Ierror = 3$.

Table 4: Results for the test functions.

#	Problem	DIRECT		DIRECT-l	
		f.eval.	p	f.eval.	p
1	Constant	9	0.00E+00	7	0.00E+00
2	Linear	475	0.76E-02	173	0.76E-02
3	Quadratic	139	0.29E-02	65	0.29E-02
4	Gomez 3	771	0.35E-03	745	0.35E-03
5	Branin	195	0.98E-03	159	0.98E-03
6	Shekel-5	155	0.84E-02	147	0.84E-02
7	Shekel-7	145	0.94E-02	141	0.94E-02
8	Shekel-10	145	0.97E-02	139	0.97E-02
9	Hartman	199	0.85E-02	111	0.85E-02
10	Hartman	571	0.89E-02	295	0.89E-02
11	Goldman-Price	191	0.30E-02	115	0.30E-02
12	Sixhump camel back	285	0.48E-03	191	0.48E-03
13	Shubert	2967	0.50E-02	2043	0.50E-02

These result show that `DIRECT-l` should be used for lower dimensional problems which do not have too many local and global minima.

References

[1] R. G. Carter. Private communications.

[2] R.G. Carter, J.M. Gablonsky, A. Patrick, C.T. Kelley, and O.J. Esslinger. Algorithms for noisy problems in gas transmission pipeline optimization. Technical Report CRSC-TR00-10, Center for Research in Scientific Computation, North Carolina State University, May 2000.

[3] T. D. Choi, P. Gilmore, O. J. Eslinger, C. T. Kelley, A. Patrick, and J. M. Gablonsky. Iffco: Implicit Filtering for Constrained Optimization, Version 2. Technical Report CRSC-TR99-23, Center for Research in Scientific Computation, North Carolina State University, July 1999. available by anonymous ftp from math.ncsu.edu in pub/kelley/iffco/ug.ps.

[4] S. E. Cox, R.T. Haftka, C. A. Baker, B. Grossman, W. H. Mason, and L. T. Watson. Global multidisciplinary optimization of a high speed

civil transport. In *Proc. Aerospace numerical Simulation Symposium '99*, pages 23–28, Tokyo, Japan, June 16-18 1999.

[5] S. E. Cox, R.T. Haftka, C. A. Baker, B. Grossman, W. H. Mason, and L. T. Watson. Global optimization of a high speed civil transport configuration. In *Proc. 3rd World Congress of Structural and Multidisciplinary Optimization*, Buffalo, NY, 1999.

[6] Evin J. Cramer. Using approximate models for engineering design. In *Proceedings of the 7th AIAA/USAF/NASA/ISSMO Symposium on Multidisciplinary Analysis and Optimization*, pages 140–147, St. Louis, MO, September 2-4 1998. AIAA-98-4716.

[7] L.C.W. Dixon and G.P. Szegö. The Global Optimisation Problem: An Introduction. In L.C.W. Dixon and G.P. Szegö, editors, *Towards Global Optimization 2*, volume 2, pages 1–15. North-Holland Publishing Company, 1978.

[8] J. M. Gablonsky. An implemention of the DIRECT Algorithm. Technical Report CRSC-TR98-29, Center for Research in Scientific Computation, North Carolina State University, August 1998.

[9] J.M. Gablonsky. *Modifications of the DIRECT Algorithm.* PhD thesis, North Carolina State University, 2001. Pending.

[10] J.M. Gablonsky and C.T. Kelley. A locally-biased form of the DIRECT algorithm. Technical Report CRSC-TR00-31, Center for Research in Scientific Computation, North Carolina State University, December 2000. To appear in Journal of Global Optimization.

[11] S. Gomez and A. Levy. The tunneling method for solving the constrained global optimization problem with several non-connected feasible regions. In A. Dold and B. Eckmann, editors, *Lecture Notes in Mathematics 909*, Nonconvex Optimization and Its Applications, pages 34–47. Springer-Verlag, 1982.

[12] W. Huyer and A. Neumaier. Global optimization by multilevel coordinate search. *J. Global Optim.*, 14(4):331–355, 1999.

[13] E. Janka. Vergleich Stochastischer Verfahren zur Globalen Optimierung. Diplomarbeit, Universität Wien, 1999.

[14] D. R. Jones. The DIRECT global optimization algorithm, 1999. To appear in The Encyclopedia of Optimization.

[15] D. R. Jones, C. D. Perttunen, and B. E. Stuckmann. Lipschitzian optimization without the lipschitz constant. *Journal of Optimization Theory and Applications*, 79:157, October 1993.

[16] Yong Yao. Dynamic Tunneling Algorithm for Global Optimization. *IEEE Transactions on Systems, Man, and Cybernetics*, 19(5), September / October 1989.
